# Acute ST-segment elevation myocardial infarction after amoxycillin-induced anaphylactic shock in a young adult with normal coronary arteries: a case report

**DOI:** 10.1186/1471-2261-5-6

**Published:** 2005-02-25

**Authors:** Aristofanis Gikas, George Lazaros, Kalliopi Kontou-Fili

**Affiliations:** 1 Health Center of Salamis, Salamis, Greece; 2 Cardiology Department, 'Elpis' General Hospital of Athens, Athens, Greece; 3 Department of Allergology and Clinical Immunology, 'Laiko' General Hospital of Athens, Athens, Greece

## Abstract

**Background:**

Acute myocardial infarction (MI) following anaphylaxis is rare, especially in subjects with normal coronary arteries. The exact pathogenetic mechanism of MI in anaphylaxis remains unclear.

**Case presentation:**

The case of a 32-year-old asthmatic male with systemic anaphylaxis, due to oral intake of 500 mg amoxycillin, complicated by acute ST-elevation MI is the subject of this report. Following admission to the local Health Center and almost simultaneously with the second dose of subcutaneous epinephrine (0.2 mg), the patient developed acute myocardial injury. Coronary arteriography, performed before discharge, showed no evidence of obstructive coronary artery disease. In vivo allergological evaluation disclosed strong sensitivity to amoxycillin and the minor (allergenic) determinants of penicillin.

**Conclusion:**

Acute ST-elevation MI is a rare but potential complication of anaphylactic reactions, even in young adults with normal coronary arteries. Coronary artery spasm appears to be the main causative mechanism of MI in the setting of "cardiac anaphylaxis". However, on top of the vasoactive reaction, a thrombotic occlusion, induced by mast cell-derived mediators and facilitated by prolonged hypotension, cannot be excluded as a possible contributory factor.

## Background

Acute myocardial infarction (MI) complicating anaphylaxis induced by drugs or other chemicals is uncommon and only sporadic cases have been reported [1-9]. The underlying pathogenetic mechanisms have not been fully elucidated. The case of a 32-year-old man with normal coronary arteries, who developed acute MI following amoxycillin-induced anaphylaxis, is the subject of this report. The underlying pathogenetic mechanisms are also discussed and the relative literature is reviewed.

## Case presentation

A 32-year-old man was admitted to the emergency room of the local Health Center because of anaphylaxis, which developed 2 hours and 15 minutes after the ingestion of amoxycillin (500 mg), prescribed by his dentist. Prodromal signs of anaphylaxis (flushing, pruritus, warmth, urticaria) reportedly occurred about 15 minutes before the onset of symptoms from other systems. Ten days earlier the patient, an asthmatic since childhood, had completed a 4-day course of amoxycillin (500 mg TID) without any side effects. Apart from obesity, there were no other risk factors for coronary artery disease.

**Figure 1:**
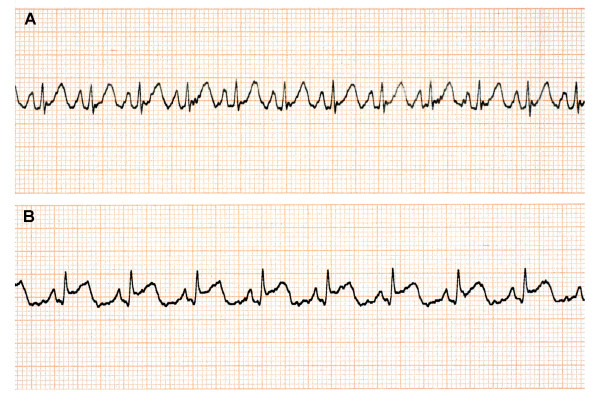
Lead II monitor strip recorded shortly after admission (A) and at the chest pain onset (B).

On admission the patient was in acute distress. He was complaining of dizziness, blurred vision, dyspnea and abdominal pain. Initial examination revealed an obese man (Weight = 130 kg, Body Mass Index = 38 kg/m^2^) with generalized erythema, angioedema, cyanosis and diffuse wheezing; the systolic blood pressure was 70 mmHg and the pulse rate 120 bpm. The patient was then connected to a cardiac monitor, which showed sinus tachycardia (approximately 140 bpm) without ST-segment and T wave abnormalities (Fig. 1A). Pulse oxymetry demonstrated an oxygen saturation (SpO_2_) of 90%.

Epinephrine (0.3 mg or 0.3 ml of a dilution 1:1000) was injected subcutaneously (SC); dimethindene (4 mg) and hydrocortizone (500 mg) were administered intravenously (IV) in slow infusion. Nebulized salbutamol and supplemental oxygen were given as well. Normal saline with 50 mg ranitidine hydrochloride and Ringer's solution were infused through separate intravenous lines.

**Figure 2:**
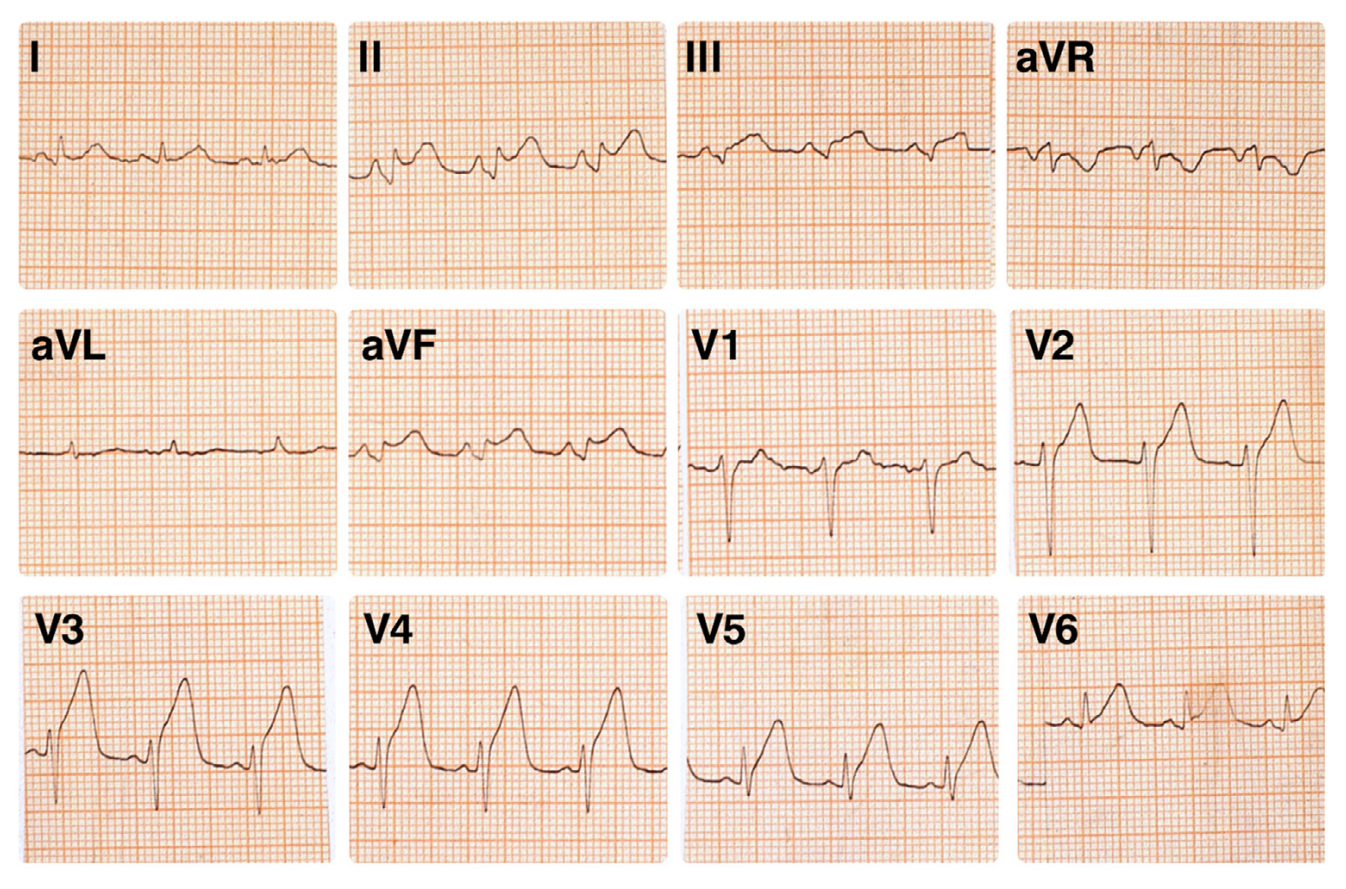
ECG recorded during chest pain.

Due to unsatisfactory clinical response (persistence of hypotension and tachycardia, despite improvement of the pulmonary signs) a second dose of epinephrine (0.2 mg) was given SC 20 minutes later. Almost simultaneously with the administration of the second dose of epinephrine, ST-segment elevation appeared on the monitor (Fig. 1B) and the patient complained of substernal chest pain. A 12-lead electrocardiogram (ECG) showed ST segment elevation in leads II, III, aVF, and V_3 _to V_6 _(Fig. 2). A single dose of 325 mg acetylsalicylic acid was given per os; heparin (5000 UI) and pethidine hydrochloride (25 mg) were administered IV. An hour later the patient was hemodynamically stable. The arterial blood pressure was 125/90 mmHg and SpO2 rose to 96%. However, the chest pain persisted and nitroglycerin titrated at a dose of 25 μg/min was infused IV, without complete symptom relief. At this point the patient was transferred to the Coronary Care Unit (CCU) of the nearest general hospital.

**Figure 3:**
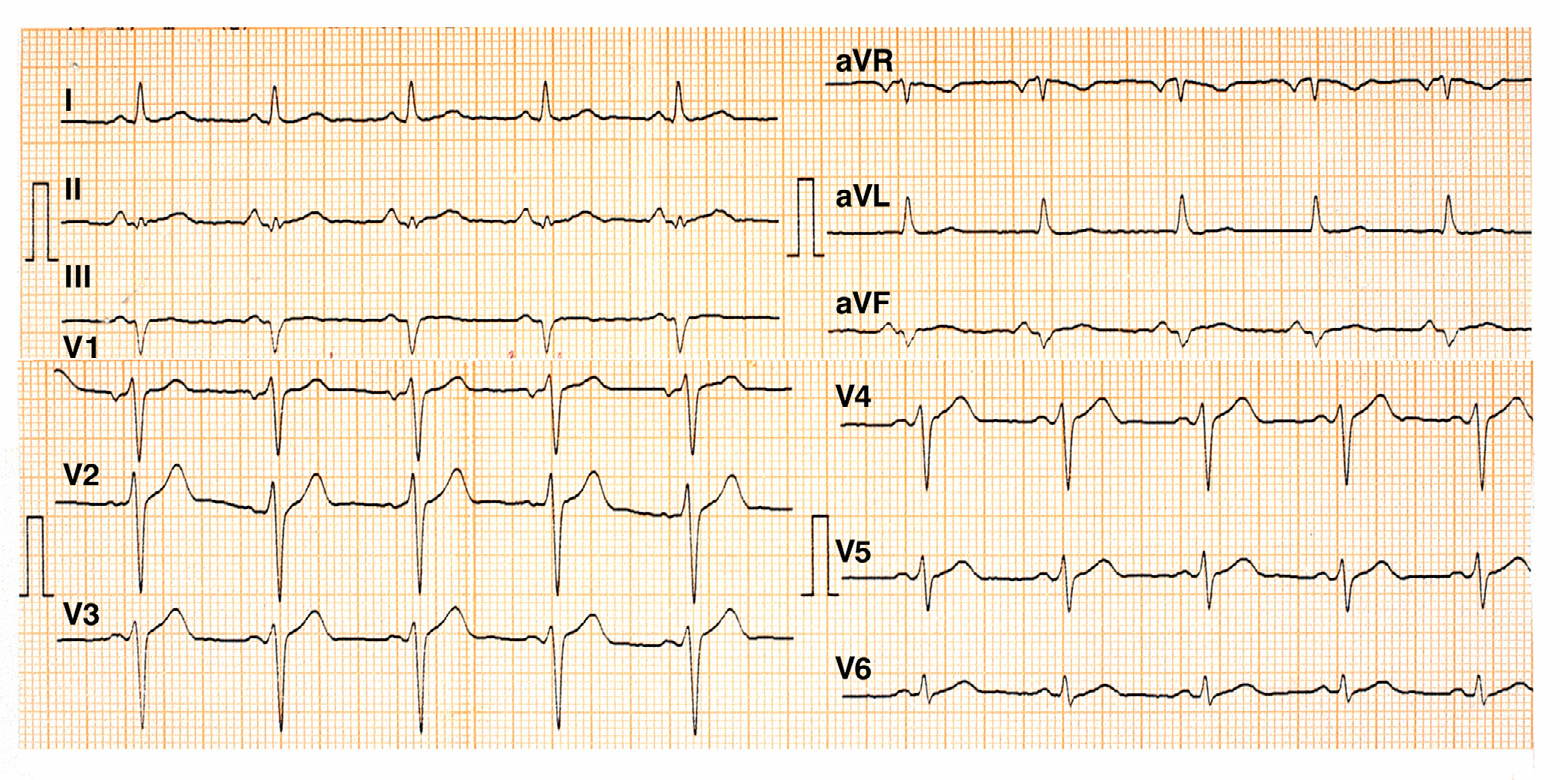
ECG recorded on hospital day 4^th^.

**Figure 4:**
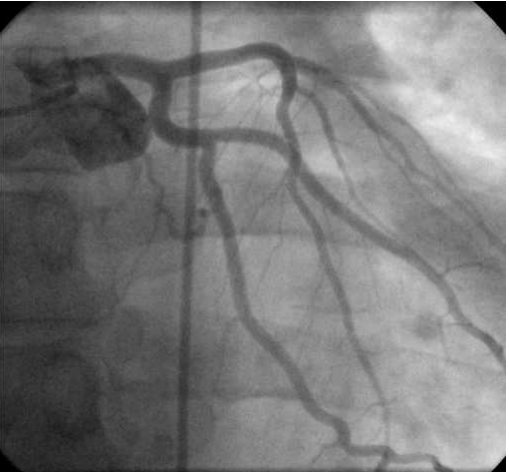
Left coronary angiogram.

**Figure 5:**
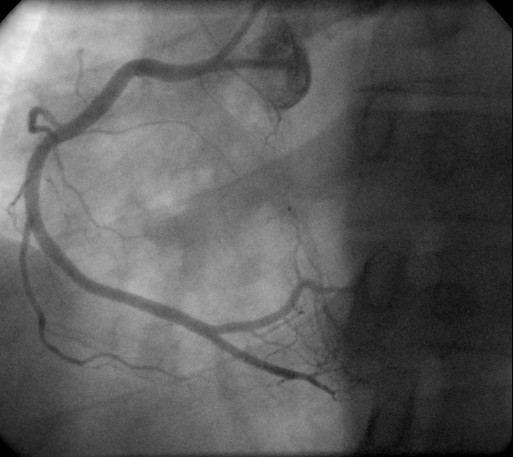
Right coronary angiogram.

Treatment in the CCU included thrombolysis with reteplase – administered according to the standard protocol – 2 hours after the onset of chest discomfort, which according to the established clinical and electrocardiographic criteria was considered successful [10]. The peak levels of serum troponin I and creatine phosphokinase were 45.5 ng/ml (normal = 0–2 ng/ml) and 575 U/L (normal = 25–195 U/L), respectively; while the MB fraction was 77 U/L (normal = 0–24 U/L). The rest of the laboratory results, including serum cholesterol, LDL, HDL, Lpa and triglycerides, were within normal limits. An ECG performed before discharge showed complete loss of potentials in leads III and aVF and partial loss of potentials with a small q wave in lead II (Fig. 3). An echocardiographic study performed on the 5^th ^hospital day showed preserved systolic function (ejection fraction 60%) without wall motion abnormalities. Left (Fig. 4) and right (Fig. 5) coronary angiography showed no evidence of obstructive coronary artery disease; left ventriculography was normal as well. The patient recovered completely and was discharged a week after admission. He was referred to the adult Allergology Center of our area performing diagnostic work up for drug allergy.

Prick skin tests (PST) were performed using penicilloyl polylysine (PPL), and minor determinant mixute (MDM) [supplied as Allergopen by Allergopharma (Reinbeck, Germany)]; amoxycillin, ampicillin and cefamandole solutions were also used (concentration 20 mg/ml) for PST. PST were strongly positive [4+ reaction (on a scale of 1–4) i.e. wheal >5 mm in diameter with pseudopodes, with no reaction at all to the diluent control] to amoxycillin and ampicillin. Intradermal tests were performed only to PPL, MDM (dilution 1/10) and cefamandole (0.2 mg/ml and 2 mg/ml) and resulted in strongly positive reaction (4+) only to the MDM. Circulating specific IgE to penicillin V, penicillin G, amoxycillin, ampicillin and cefaclor was not demonstrable by CAP (Pharmacia, Sweden).

## Discussion

Anaphylactic reactions may trigger cardiovascular events, including MI [1-9], primarily in subjects with underlying ischemic heart disease [1,2,4,7,9] and rarely in individuals with normal coronary arteries [3,5,6,8]. Cardiovascular complications in the setting of anaphylaxis rarely occur in subjects less than 35 years old [11].

Acute coronary syndromes following antibiotic-induced anaphylaxis is uncommon and only few cases have been reported so far [1,4,12-14]. To our knowledge, only one case of amoxycillin-induced anaphylaxis complicated with acute MI has been documented in a 62-year-old man with underlying coronary artery disease [4].

Evidence that the human heart is the target as well as the site of anaphylaxis is constantly growing. A number of studies by Marone et al [15,16] and others [17] have demonstrated mast cells within the human heart; they are strategically located perivascularly, in close proximity to myocytes, in the arterial intima and the shoulder region of atheromas. Cardiac mast cells express on their surface high affinity receptors for IgE (FcεRI) and also C5a receptors [18]. Mast cells activation through immunological and non-specific (non-immunological) stimuli leads to the release of a variety of vasoactive and pro-inflammatory mediators, preformed or de novo synthesized (histamine, prostaglandins, leukotrienes, enzymes, cytokines and others) [15,19]. Coronary spasm, which is proposed as the main underlying mechanism of allergy-induced coronary syndromes [5,11,13,14,20,21], can be caused, directly or indirectly, by potent vasoactive mast cell-derived mediators such as histamine, prostaglandin D2, thromboxane, cysteinyl leukotrienes [15,18,22-24]. In addition, mast cells are likely to affect coagulation and fibrinolysis at different levels through enzymes and mediators they secrete [15]. Therefore, mediators from perivascular and interstitial cardiac mast cells – as well as those reaching the heart from the pulmonary circulation – might affect coagulation, favoring platelet aggregation and thrombus formation [15,25].

In the present case, the temporal sequence of events suggests that cardiac anaphylaxis was the triggering factor of MI. We propose that prolonged coronary vasospasm induced by vasoactive and inflammatory mediators, released during anaphylaxis, was the main causative mechanism. However, in view of the persistence of chest pain following IV nitroglycerin infusion, and its complete resolution promptly after IV thrombolysis, a thrombotic vascular occlusion, on top of the vasospastic reaction, cannot be excluded. The latter is also supported by the prolonged systemic hypotension, which, as it has been emphasized in previously reported cases [26], probably caused further reduction of the myocardial perfusion, thus, favoring in situ thrombus formation and subsequent coronary artery occlusion. In acute MI cases, presenting to centers with cardiac catheterization facilities, urgent coronary arteriography appears as the management strategy of choice for the final diagnosis. However, in the setting of acute anaphylaxis, this might not be the wisest choice, particularly in cases, like the present one, in which the offending allergen had been administered orally. Moreover, continuous or delayed allergen absorption could further aggravate anaphylaxis, and a late phase reaction represents a potential risk in all anaphylactic reactions. Finally, no bibliographical data are available concerning the tolerability of IV administered contrast agents in patients who have suffered a recent episode of severe systemic anaphylaxis.

The administration of epinephrine – a life saving agent in cases of anaphylaxis – has been implicated as a cause of acute MI in a limited number of reports [27,28]. In the present case, however, it appears unlikely that exogenous epinephrine was the initiating event for the following reasons: a) the first epinephrine dose (0.3 mg) was rather low to induce significant vasoconstriction in a subject with a body weight of 130 kg, b) the mode of administration (SC) is considered the safest in this regard, c) the second dose (0.2 mg), administered at a safe interval (20 min) after the first one, does not seem to be involved since its injection coincided with the onset of chest pain, before any anticipated drug absorption. On the contrary, in the above quoted cases [27,28] the epinephrine-induced MI developed 5–15 minutes post injection. Moreover, the possibility of inadvertent intravenous administration of epinephrine appears improbable, since no blood was withdrawn in the syringe before drug injection. However, one might argue that the exogenous epinephrine could have aggravated a pre-existing coronary spasm, induced by mast cell-derived mediators. Hence, the precise effect of epinephrine in this clinical setting remains a matter of speculation.

The allergological evaluation performed in our patient showed strong sensitivity to amoxycillin and to the minor determinants, which are the allergenic epitopes associated with systemic anaphylaxis. The negative CAP results do not negate the in vivo findings, since it is well established that the in vitro techniques are not as sensitive as skin tests. Furthermore, CAP detects antibodies against the major determinants of penicillins (involved pathogenetically in penicillin-induced urticaria) and not to the minor ones, which are implicated in systemic anaphylaxis. For technical reasons, relating to both the Allergology Center and the patient's professional obligations as well as residence distance, the allergological work up was completed 6 months later; such a delay might also have contributed to the negative in vitro findings.

## Conclusion

Acute ST-elevation MI is a rare but potential complication of anaphylactic reactions, even in young adults with normal coronary arteries. Physicians should be alert for such a complication in order to diagnose it early and treat properly.

It is fairly well established that that human heart can be both, the site and the target of severe anaphylaxis; in this setting cardiac mast cells – activated and releasing multiple vasoactive mediators – play an important role in pathogenesis of cardiac complications [16-18].

In the above case, mediator-induced coronary artery spasm was the main, but probably not the exclusive causative mechanism of anaphylaxis-related MI. The thrombotic vascular occlusion, induced by inflammatory mediators and facilitated by prolonged hypotension, cannot be excluded as a possible contributory factor.

## Abbreviations

ECG = electrocardiogram; IV = intravenously; MDM = minor determinant mixute; MI = myocardial infarction; NV = normal values; PPL = penicilloyl polylysine; PST = prick skin tests; SC = subcutaneously.

## Competing interests

The author(s) declare that they have no competing interests.

## Authors' contributions

AG was responsible for the initial evaluation and management of the patient, GL was involved in the cardiological evaluation and management and, KK-F performed the allergological evaluation. All authors have equally contributed in the preparation and revision of the manuscript. All authors read and approved the final manuscript.

## Pre-publication history

The pre-publication history for this paper can be accessed here:


